# Depressive symptoms and subjective cognitive dysfunction associated with non-suicidal self-injury among Chinese adolescents: the mediating role of impulsivity and the moderating role of addictive features

**DOI:** 10.3389/fpsyt.2026.1552165

**Published:** 2026-02-06

**Authors:** Gengliang Li, Ruiqi Wang, Tiantian Fu, Shiyu Tong, Feng Tian

**Affiliations:** 1 The Second Hospital of Shanxi Medical University, Taiyuan, Shanxi, China; 2 The Second Clinical Medical College of Shanxi Medical University, Taiyuan, Shanxi, China; 3 Department of Psychiatry, the Second Hospital of Shanxi Medical University, Taiyuan, Shanxi, China

**Keywords:** addictive feature, depressive symptom, impulsivity, non-suicidal self-injury, subjective cognitive dysfunction

## Abstract

**Background:**

Major depressive disorder (MDD) and non-suicidal self-injury (NSSI) have a strong connection, and adolescents with MDD who engage in NSSI typically exhibit more severe subjective cognitive dysfunction. This study aimed to clarify how depressive symptoms are associated with subjective cognitive dysfunction in adolescents with NSSI, with a focus on the moderating role of addictive traits and impulsivity levels.

**Methods:**

260 adolescents aged 12–18 years with MDD were recruited and divided into the NSSI group (150 cases) and without NSSI group (110 cases). The subjects were evaluated using the Ottawa Self-Injury Inventory (OSI), Patient Health Questionnaire (PHQ-9), Barratt Impulsivity Scale (BIS-11), and Self-Cognitive Functional Deficits Scale (PDQ-D). The scores of the two groups were compared, and the roles of the impulsivity level and characteristics of self-injurious addictions in the relationships between subjective cognitive dysfunction and depressive symptoms were examined.

**Results:**

In comparison to the group without NSSI, the NSSI group scored significantly higher on depressive symptoms, impulsivity level, and subjective cognitive dysfunction (*p* < 0.001). Additionally, the depression scores in the group with NSSI were significantly positively correlated with the scores in addictive features, impulsivity level, and subjective cognitive dysfunction. Furthermore, impulsivity level partially mediated the increase of subjective cognitive dysfunction by depressive symptoms, with a mediation effect value of 0.118 and 95%CI=[0.004,0.249]. Conversely, there was a significant interaction term for subjective cognitive dysfunction between depressive symptoms and self-injurious addiction feature (*β* = 0.063,95%CI=[0.008,0.117]).

**Conclusion:**

Adolescents with MDD who have NSSI have more severe subjective cognitive dysfunction in the context of high impulsivity levels and the interaction between depressive symptoms and self-injurious addictive Features.

## 1 Introduction

Non-suicidal self-injury (NSSI) is a prevalent yet perplexing behavioral issue in which an individual deliberately and directly inflicts damage on one’s own bodily tissues, such as through cutting, burning, scratching, or hitting, without any intent to end their life (1). NSSI is prevalent among adolescents, with a global lifetime prevalence estimated at 22.0% (2), and an even higher lifetime prevalence of 22.3% in China (3). NSSI has a serious impact on the physical and mental health of adolescents. NSSI has been found to be associated with a variety of mental disorders, especially major depressive disorder (MDD) (4). Adolescents with comorbid NSSI and MDD typically exhibit more severe subjective cognitive dysfunction, including impairments in memory, attention, executive function, and interference control (5, 6). NSSI could be additionally linked to abnormal functional connections in the prefrontal temporal brain, according to recent neuroimaging research. For instance, NSSI behavior is substantially associated with reduced functional connectivity in the bilateral prefrontal temporal cortex in adult MDD patients, and this alteration is connected to working memory and language learning deficiencies (7). Therefore, it is necessary to study the specific pathogenesis of subjective cognitive dysfunction in patients with NSSI.

MDD is a common chronic mental illness characterized by enduring low mood, diminished energy, and anhedonia, correlated with elevated rates of morbidity, relapse, self-injury and suicide, frequently accompanied by cognitive impairment (8). While NSSI is significantly correlated with teenage major depressive disorder (MDD), a recent study revealed that 49.1% of adolescents experiencing their first episode of MDD had a prior history of NSSI (9). Longitudinal studies have also indicated that depression is the most frequent predictor of NSSI (10). One of the main reasons individuals experience NSSI is the experience of overwhelming negative feelings, but lack the ability to regulate these feelings (11). In addition, it has been shown that people with MDD with NSSI exhibit more prominent subjective cognitive dysfunction in problem reasoning, organization, attention, and working memory than people with MDD without NSSI (11, 12). Adolescents diagnosed with MDD and NSSI demonstrated significantly extended latencies and diminished amplitudes in the N1, N2, P3a, P3b, and P50 components of event-related potentials (ERPs). These findings suggest extensive deficits in memory, attention, and executive functions (6). And subjective cognitive dysfunction can amplify mild negativity into a strong, dysfunctional pattern of negative cognition that drives and sustains depressive symptoms (13).

Both the addictive feature of NSSI (14, 15) and elevated impulsivity (16, 17) have been proposed as mechanisms contributing to subjective cognitive dysfunction in individuals with MDD. NSSI has been described as having an addictive profile or function (18, 19), a repeated inability to resist the temptation to self-injury. It has been proposed that the addictive characteristics of NSSI are comparable to those of substance abuse. On the one hand, the addictive feature of NSSI causes anomalies and structural-functional changes in the brain’s amygdala circuits (14), which exacerbate cognitive dysfunction, affecting memory, attention, executive functioning, and decision-making abilities. On the other hand, NSSI addiction traits are equally positively and negatively reinforcing, perpetuating NSSI and potentially leading to the development of dependence (20). In addition, individuals may develop an addiction-like pattern of NSSI, characterized by a loss of control and marked tolerance. Despite adverse consequences, this compulsive engagement can be difficult to restrain, thereby contributing to a further decline in cognitive function (21). Whereas, impulsivity level as an individual behavioral trait includes behavioral disinhibition, risky decision-making, and delayed discounting (22). Studies have found a link between depressive symptoms and impulsivity levels (23). Emotion-based impulsive behaviors are associated with NSSI onset and cognitive-based impulsive behaviors are associated with NSSI maintenance (16). In addition, there are interactions between impulsivity levels and cognitive functioning, such as a study of MDD adolescents that showed that the impulsivity trait they exhibited was associated with impaired cognitive flexibility (17). At the same time, the cognitive dysfunction may be underpinned by neural mechanisms common to both impulsivity and addiction. Supporting this, the P300 component (specifically P3b), a neural marker of impulsivity originating from the prefrontal cortex, has been identified as a key mediator in predicting improvement in NSSI behaviors, highlighting its critical role in treatment response (24). Furthermore, there are notable distinctions between the cognitive features of various self-injurious behaviors (such as NSSI and SA): the SA group exhibits more severe deficits in working memory and executive function, whereas the NSSI group’s cognitive function is strongly associated with borderline personality traits and rumination tendencies (25).The findings suggest that impulsivity may modulate the relationship between depressive symptoms and cognitive functioning, influencing cognitive performance in persons with MDD.

As a result, we propose that impulse level may be one of the mediators mediating the relationship between symptoms of depressive disorder and subjective cognitive dysfunction, and that NSSI addiction features may co-regulate the level of subjective cognitive dysfunction in MDD adolescents via interaction with depression symptoms. Although the above studies have shown that NSSI is strongly associated with depressive symptoms, subjective cognitive dysfunction, impulsivity levels, and addictive features, there are no studies that have explored the relationship between the four and the underlying mechanisms. The current study will examine the mechanisms influencing subjective cognitive dysfunction in adolescents with MDD with NSSI and evaluate the differences in depressive symptoms, impulsivity levels, and cognitive performance between adolescents with MDD and those without NSSI. We anticipate that this research will improve knowledge about NSSI and lay a solid evidence-based basis for the prevention and treatment of cognitive dysfunction in adolescents with MDD.

## 2 Participants and methods

### 2.1 Participants

Between January 2024 and September 2024, 260 adolescents with MDD who were receiving treatment in the psychiatry department of the Second Hospital of Shanxi Medical University were selected as the study subjects through the use of questionnaires and interviews, as per the convenience sampling principle. Inclusion criteria: (1) Age between 12 and 18 years old; (2) Meet the diagnostic criteria of MDD and NSSI in the Diagnostic and Statistical Manual of Mental Disorders, Fifth Edition (DSM-5). Exclusion criteria: (1) Patients who also experienced severe agitation, defiance, and non-cooperation; (2) Patients who also suffered from other psychiatric disorders, serious somatic diseases, or organic brain diseases.

The study [2024YX No.244] was approved by the Ethics Committee of the Second Hospital of Shanxi Medical University and all patients and their guardians gave their informed consent.

### 2.2 Methods

#### 2.2.1 Measures

General Information Questionnaire The sociodemographic and clinical characteristics of the recruited teenagers with MDD were examined using a self-administered general information questionnaire that collected data on age, gender, education, years of schooling, length of illness, and treatment history.

Patient Health Questionnaire-9 (PHQ-9) (26) This questionnaire, as one of the clinical assessment scales for depression, not only has the function of assisting the diagnosis of depressive disorders, but also can be used for the assessment of the severity of the disease. The scale includes 9 items with ratings ranging from 0 to 3, for a total score of 0-27. A PHQ-9 score of less than 5 indicates no overt signs of depression, 5–9 indicates mild depression, 10–14 indicates moderate depression, 15–19 indicates fairly severe depression, and a score of 20 or above indicates severe depression. In this study, the scale’s Cronbach’s alpha coefficient was 0.91, indicating strong internal consistency.

The Ottawa Self-Injury Questionnaire (OSI) (27) This questionnaire is designed to assess the occurrence, frequency, characteristics, functions, and addictive features of non-suicidal self-injury (NSSI). In this study, the Addiction Features subscale of the OSI was employed to operationally define and measure addictive features of NSSI. This subscale captures core dimensions of behavioral addiction, including craving, loss of control, difficulty reducing or ceasing NSSI (withdrawal), and escalation of behavior (tolerance). The OSI has demonstrated adequate validity and reliability in both adolescent inpatient (27) and non-clinical samples (28). Additionally, the Behavioral Functioning subscale was administered to evaluate psychological motivations for engaging in NSSI.

Barratt Impulsivity Scale (BIS-11) (29) This scale is one of the most commonly used impulsivity evaluation measures and consists of 3 dimensions: attentional impulsivity, motor impulsivity and non-planning impulsivity. A Likert scale is employed (1 “never” to 5 “often”), with higher total scores indicating higher levels of impulsivity and higher dimension scores suggesting more impulsive performance in that area. In the present investigation, the Cronbach’s alpha coefficient for this questionnaire was 0.85.

Perceived Deficits Questionnaire-Depression (PDQ-D) (30) The PDQ-D contains 20 items, each rated on a scale of 1 to 5, with a total score of 20 to 100. Higher scores indicate more severe subjective cognitive dysfunction. The scale has been widely used to assess adolescents’ subjective cognitive functioning (31), and demonstrated good internal consistency in this study, with a Cronbach’s alpha coefficient of 0.96.

#### 2.2.2 Quality control

All diagnoses were made by two or more attending psychiatrists and confirmed by consensus. All assessments will be conducted by a professional psychometrician in a quiet environment, taking 20 minutes to 50 minutes. Upon completion, all questionnaires were checked for missing items, which were completed on site. Data were entered into SPSS by two researchers, verified individually and invalid questionnaires removed.

#### 2.2.3 Statistical analysis

Data were analyzed using SPSS 27.0. Categorical data were presented as frequency and percentage (%), and comparisons between groups were performed using the *χ2* test. Normally distributed data were expressed as mean ± standard deviation ( $\bar{x}$ ± *s*), and the independent samples t-test was used to compare two groups. Pearson correlation was used to assess the relationship between depressive symptoms, impulsivity levels, addictive behaviors, and subjective cognitive dysfunction in MDD adolescents with NSSI. The PROCESS V4.0 macro was used for the moderated mediation analysis, employing standardized regression coefficients (adjusted for gender, age, and education level) as path coefficients. The significance of the indirect effects was tested using a bootstrap approach with 5,000 resamples to generate bias-corrected 95% confidence intervals. A difference was considered statistically significant at *p* < 0.05.

## 3 Results

### 3.1 Demographic and clinical characteristics

A total of 260 adolescents with MDD were included, with 150 (114 females, mean age 15.54 ± 1.57 years) in the MDD with NSSI group and 110 (78 females, mean age 15.85 ± 1.59 years) in the MDD without NSSI group. Age, years of education, sex, and academic degree did not significantly differ between these two groups of participants (all *p*>0.05); however, the MDD with NSSI group’s scores for depression severity, impulsivity, and subjective cognitive dysfunction were significantly higher than those in the MDD without NSSI group (all *p* < 0.05). Refer to **Table 1**.

**Table 1:** Demographic and clinical characteristics of adolescents with MDD [ $\bar{x}$± *s*, *n*(%)].

Basic characteristics	MDD with NSSI group(*n* = 150)	MDD without NSSI group (*n* = 110)	*t*/*χ2*	*p*
Age	15.54 ± 1.57	15.85 ± 1.59	1.590	0.113
Sex			0.852	0.356
Male	36 (24.0)	32 (29.1)		
Female	114 (76.0)	78 (70.9)		
Education years	9.57 ± 1.64	9.91 ± 1.67	1.651	0.100
Academic degree			4.089	0.129
Junior high school	72 (48.0)	39 (35.4)		
Senior high school	70 (46.7)	64 (58.2)		
University	8 (5.3)	7 (6.4)		
Depression score	16.32 ± 5.68	11.45 ± 5.83	-6.746	<0.001
Impulsivity	58.67 ± 11.49	50.96 ± 11.55	-5.333	<0.001
Exercise impulsivity	18.01 ± 5.67	15.70 ± 5.57	-3.267	0.001
Attentional impulsivity	15.77 ± 3.98	12.23 ± 4.14	-6.987	<0.001
Purposeless impulsivity	24.87 ± 6.10	23.04 ± 5.62	-2.480	0.014
Subjective cognitive dysfunction	44.12 ± 16.84	27.82 ± 16.93	-7.695	<0.001

MDD, major depression disorder; NSSI, nonsuicidal self-injury.

### 3.2 Characteristics of NSSI in adolescents with MDD

In this study, 57.7% of adolescents with MDD met the diagnostic criteria for NSSI and reported varying degrees of NSSI behaviors in the past month. In the behavioral questionnaire, 28 cases (18.7%) adopted only one form of self-injury and 122 cases (81.3%) had more than one form of self-injury. The most prevalent forms of self-injury were “cutting” (72%), “stabbing a body part with a sharp object” (42%), and “hitting” (37%). The vast majority of adolescents (94.9%) perceived NSSI as a strategy to alleviate stress or negative emotions. Scores on the NSSI functioning assessment showed social influence at 5.43 ± 4.27, internal emotion regulation at 8.31 ± 4.39, and external emotion regulation at 11.55 ± 4.27.

### 3.3 Correlation analysis of various psychological factors in adolescent MDD patients with NSSI

The findings demonstrated a substantial positive association (all *p* < 0.05) between the patients’ ratings for subjective cognitive dysfunction, impulse levels, addictive features, and depressive symptoms. Refer to **Table 2**.

**Table 2:** Correlation analysis of various psychometric indicators in MDD patients with NSSI.

Indicators	Scores ( $\bar{x}$±*s*)	Depressive symptoms	Addictive features	Impulsivity	Subjective cognitive dysfunction
Depressive symptoms	16.32 ± 5.68	1			
Addictive features	13.08 ± 5.90	0.408**	1		
Impulsivity	58.67 ± 11.49	0.175*	0.239**	1	
Subjective cognitive dysfunction	44.12 ± 16.84	0.601**	0.354**	0.384**	1

*p < 0.05, **p < 0.01.

### 3.4 Mediating role of impulsivity level

Depressive symptoms were the independent variable, subjective cognitive dysfunction was the dependent variable, and gender, age, and education were the control factors in Process’s Model 4. **Table 3** demonstrates that depressive symptoms were directly associated with impulsivity level (*β* = 0.323, *p* < 0.05) and with subjective cognitive dysfunction (*β* = 1.588, *p* < 0.001). High levels of impulsivity were associated with more severe subjective cognitive dysfunction, and the relationship between impulsivity levels and subjective cognitive dysfunction was statistically significant (*β* = 0.366, *p* < 0.001). The indirect effect of depressive symptoms on subjective cognitive dysfunction through impulsivity levels was significant: INDIRECT effect = 0.118, SE = 0.062, 95%CI = [0.004, 0.249].

**Table 3:** Regression results for conditional direct effect and conditional indirect effects.

Variables	*β*	SE	*t*	*p*	LLCI	ULCI	*R^2^*	*F*
Impulsivity							0.123	5.088
Depressive symptom	0.323	0.159	2.025	0.045	0.008	0.637		
Subjective cognitive dysfunction							0.481	26.665
Depressive symptom	1.588	0.183	8.691	0.000	1.227	1.950		
Impulsivity	0.366	0.094	3.892	0.000	0.180	0.551		

### 3.5 Moderating role of self-injurious addiction features

**Figure 1:**
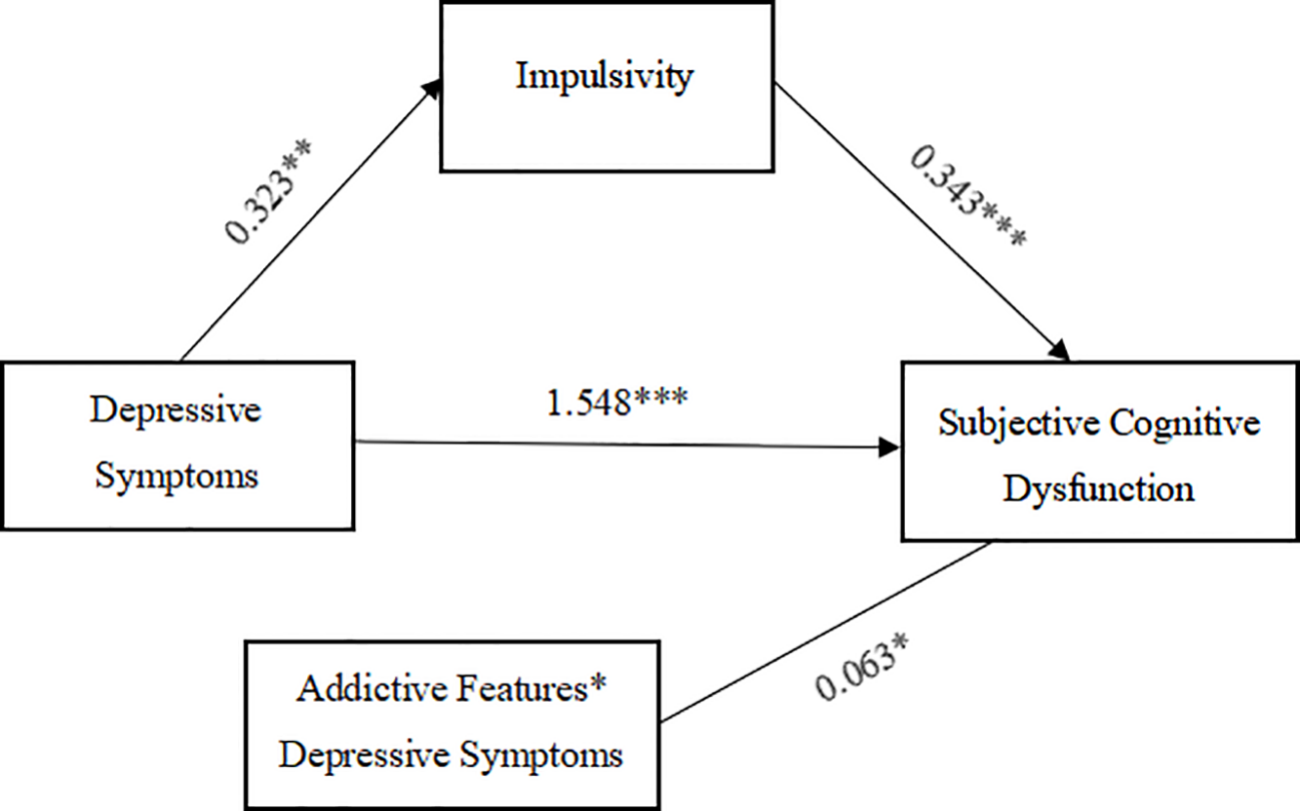
Models of mediated regulation of impulsivity levels, addiction characteristics in depressive symptoms and subjective cognitive dysfunction in MDD adolescents with NSSI. *p < 0.05, **p < 0.01, ***p < 0.01. Covariates (gender, age, literacy) are not plotted to increase readability but are included in the model.

After determining that impulsivity level mediates the relationship between depressive symptoms and subjective cognitive dysfunction, we used model 5 in Process to test for moderating effects. To avoid multicollinearity, all predictor variables were standardized and gender, age and education were accounted for. The interaction term between depressive symptoms and self-injurious addiction features was a significant predictor of subjective cognitive dysfunction (*β* = 0.063, SE = 0.028, *p* < 0.05, 95%CI = [0.008,0.117]), and the moderating effect of self-injurious addiction traits was significant, indicating the presence of moderated mediation, as shown in the model in **Figure 1**.

**Figure 2:**
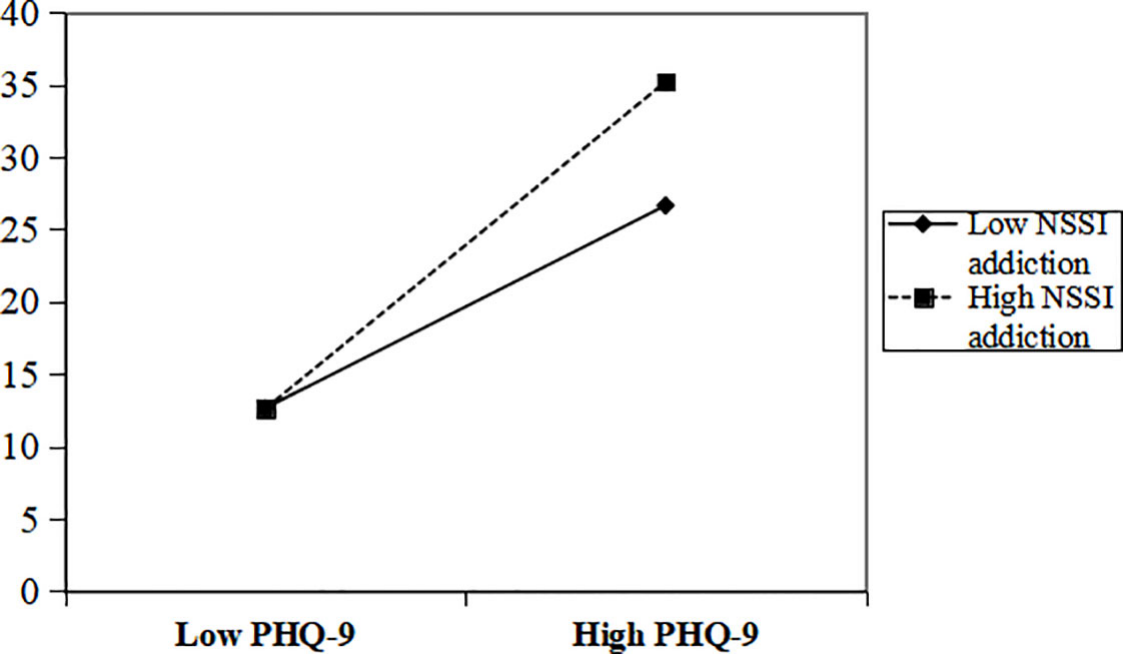
Moderating role of addiction characteristics in the relationship between depressive symptoms and subjective cognitive dysfunction at different levels of depression.

To further illustrate the moderating role of self-injurious addiction traits, a basic slope test was conducted (**Figure 2**). The results showed that depressive symptoms were significantly predictive of subjective cognitive dysfunction when scores on the self-injury addiction trait were high (*B* = 1.178,SE=0.240,*t* = 4.908,*p* < 0.001,95%CI=[0.703,1.653]), and that depressive symptoms were significantly predictive of subjective cognitive dysfunction when scores on the self-injury addiction trait were low (*B* = 1.919,SE=0.268,*t* = 7.156,*p* < 0.001, 95%CI=[1.389,2.449]).

## 4 Discussion

This study analyzed a model of how depressive symptoms, impulsivity levels, and NSSI addiction features interact in relation to subjective cognitive dysfunction in adolescents. It was discovered that depressive symptoms had a significant positive correlation with subjective cognitive dysfunction, which was partially influenced by impulsivity levels, and that depressive symptoms were also a significant predictor of subjective cognitive dysfunction in adolescents with MDD through their interaction with NSSI addiction features. Further discussion and interpretation are provided based on the study’s main findings.

According to the study’s findings, 76.0% of the 260 adolescents with MDD who participated in the study were female, and 57.7% of them reported more than five episodes of NSSI in the previous year and varying degrees of NSSI in the previous month. This high prevalence suggests that NSSI is a severe issue among Chinese adolescents with MDD, and that immediate action must be taken to address it. NSSI is more common in the female group. This result is consistent with the findings of Iswanti et al. (32), which may be related to the fact that different genders choose distinct emotion-regulating strategies for their adolescence. Adolescents of various genders may use different emotion control techniques, which could explain why NSSI is more prevalent in the female group (33). In addition, data from this study showed that 81.3% of adolescents in the MDD group with NSSI reported multiple NSSI behaviors at the same time, with cutting (72%), stabbing body parts with sharp objects (42%), and hitting (37%) being the most common NSSI modalities reported, which is similar to a previous study (34). A recent cross-sectional study indicated that experiences of bullying are the most significant predictor of non-suicidal self-injury (NSSI) behavior in adolescents (32). This study found that 81.3% of patients with NSSI utilized multiple self-harm methods, indicating that NSSI may serve as a maladaptive coping strategy for negative social experiences. According to the NSSI Functionality Scale, the primary function of NSSI is emotion regulation, exemplified by the release of unbearable tension and the relief of sadness or other negative feelings, indirectly demonstrating that NSSI is a strategy and method for adolescents to cope with negative emotions (35, 36).

Our findings demonstrated that, in comparison to the MDD without NSSI group, adolescents in the MDD group with NSSI had considerably higher scores on depressive symptoms and subjective cognitive dysfunction, as well as significantly different scores on all subscales and overall scores of the Impulsivity Questionnaire (BIS-11). Evidence from both community and hospital-based samples indicates that adolescents with NSSI exhibit elevated levels of impulsivity (37, 38), and impulsivity has been linked to more severe depression (39). These findings are consistent with our study’s findings that impulsivity may be a feature of the NSSI clinical population (40). Additionally, consistent with theoretical models, negative emotions and stimuli are often associated with the emergence of impulsive behavior (41).

This study revealed a direct and positive correlation between depressive symptoms and subjective cognitive dysfunction. This relationship may be underpinned by abnormalities in the monoamine neurotransmitter system. According to the “Basic Emotion Tricolor Model” proposed by Gu et al. (42), the three monoamine neurotransmitters—dopamine (DA), serotonin (5-HT), and norepinephrine (NE)—map onto three fundamental emotional experiences: reward, punishment, and stress, respectively. The more severe subjective cognitive dysfunction seen in adolescents with MDD accompanied by NSSI in this study is consistent with the hypothesis that depressive symptoms may be associated with an imbalance in these neurotransmitter systems, leading to impairments in cognitive areas such as working memory and executive function.

Additionally, the relationship between subjective cognitive dysfunction and depression symptoms was in part mediated by impulsivity levels. In other words, depressive symptoms are not only directly and positively associated with subjective cognitive dysfunction, but there may also exist an indirect pathway via impulsivity. Individuals with higher impulsivity tend to exhibit increased behavioral disinhibition and impulsive decision-making (43). We postulate that disinhibition may reduce an individual’s ability to suppress dominant behaviors, potentially leading to a tendency to respond to internal or external stimuli without fully evaluating potential adverse consequences (44). Moreover, although impulsive decision-making is unlikely to yield positive outcomes, such behavior may be perceived as highly rewarding in certain contexts. For instance, individuals who engage in non-suicidal self-injury (NSSI) may seek rapid emotional relief while perceiving the negative consequences as less significant (45). It has been proposed that depressed patients with impulsive traits may display irrational beliefs, rigid thinking patterns, and poor problem-solving abilities (17). Furthermore, adolescents with depression who engage in NSSI often exhibit various negative cognitions, such as low self-esteem, low self-efficacy, and self-criticism—and these cognitions may be more pronounced in the presence of higher impulsivity.

Our study further revealed that features of NSSI addiction moderate the relationship between depressive symptoms and subjective cognitive dysfunction. Specifically, higher levels of NSSI addictive traits were found to strengthen the association between depressive symptoms and subjective cognitive dysfunction, whereas lower levels exerted only minimal influence. In the present sample, 77.3% of adolescents with major depressive disorder (MDD) engaged in NSSI behaviors marked by significant addictive characteristics. Those scoring higher on the NSSI addiction profile reported more frequent self-injurious thoughts and acts, and demonstrated a greater tendency to regulate negative emotions through NSSI—a pattern consistent with previous reports (28). Moreover, prolonged engagement in repetitive NSSI may alter neural reward pathways, a notion supported by theories emphasizing dopamine’s role in reward processing (42).

The addictive pattern of non-suicidal self-injury is defined by a diminished capacity to control persistent self-injurious behaviors, which are typically followed by feelings of relaxation or euphoria. These pleasurable states reinforce intense cravings, which are associated with individuals reengaging in self-injury to reexperience a psychological “high” (28, 46). This pattern can be operationalized through core behavioral addiction features—such as salience, tolerance, and craving—which extend beyond simple behavioral repetition. Some individuals develop a pathological reliance on NSSI, often using it to achieve immediate emotional relief despite awareness of long-term adverse outcomes (15). Importantly, although emotional dysregulation may initiate NSSI as a coping strategy, the addictive pattern is principally maintained by positive reinforcement—the active pursuit of euphoria—rather than purely by negative reinforcement aimed at distress reduction. Therefore, early mental health interventions are critical for adolescents exhibiting pronounced NSSI addiction features, not only to address self-injurious behaviors but also to potentially mitigate associated cognitive dysfunction.

This study has several limitations. First, the cross-sectional design precludes causal inferences between subjective cognitive dysfunction and features of NSSI addiction or impulsivity in adolescents with MDD. Second, the assessment of subjective cognitive dysfunction relied solely on self-reported measures without objective neuropsychological tests, which may affect the accuracy of the findings. Third, the absence of biological indicators limited our ability to elucidate underlying mechanisms. Furthermore, the use of a single-center Chinese clinical sample may limit the generalizability of the results to other populations with different cultural backgrounds or healthcare systems. Finally, the relatively small sample size may increase the potential for bias. Future research should incorporate objective neuropsychological assessments and biomarkers, along with multi-phase follow-up data in large-scale, multicenter studies, to further clarify the mechanisms by which depressive symptoms influence subjective cognitive dysfunction in adolescents with NSSI and to inform targeted interventions.

This study is the first to examine the mediating and moderating roles of impulsivity levels and NSSI addiction features in the relationship between depressive symptoms and subjective cognitive dysfunction in adolescents. We discovered that the link between various levels of depressive symptoms and subjective cognitive dysfunction can be indirectly influenced by impulsivity, which may be one of the clinical aspects of MDD adolescents with NSSI. The characteristics of addiction can strengthen the relationship between depressive symptoms and subjective cognitive dysfunction and also serve as a defining feature of recurrent self-harm. By providing a partial explanation of the mechanisms underlying subjective cognitive dysfunction in MDD adolescents with NSSI and enabling the targeting of early interventions and treatments, these findings expand our understanding of the impact of impulsivity levels and addictive features on subjective cognitive dysfunction in adolescents.

## Data Availability

The raw data supporting the conclusions of this article will be made available by the authors, without undue reservation.
